# Bioinformatics analysis of pyroptosis-related differentially expressed genes in sepsis and diabetes mellitus

**DOI:** 10.1371/journal.pone.0352310

**Published:** 2026-06-25

**Authors:** Mengxuan Liu, Haiyan Song, Can Zhang, Jiaxin Li, Meng Han, Ying Li

**Affiliations:** Jinan Third People’s Hospital, Jinan, Shandong, People’s Republic of China; Noorda College of Osteopathic Medicine, UNITED STATES OF AMERICA

## Abstract

Sepsis and diabetes mellitus (DM) are major global health issues with high morbidity and mortality, necessitating new molecular insights for improved treatments. In this study, pyroptosis-related differentially expressed genes (PRDEGs) linked to both diseases were identified through bioinformatics analyses of GEO datasets (GSE28750 and GSE55098). These analyses included differential expression analysis, GO/KEGG pathway enrichment, gene set enrichment analysis (GSEA), protein-protein interaction (PPI) network construction, and peripheral blood immune cell composition analysis. Seven PRDEGs (*GZMA, GZMB, MMP9, LCN2, ANXA3, PRF1,* and *CAMP*) were identified, involved primarily in cytolysis and the IL-17 signaling pathway. GSEA indicated transcriptional changes in the IL-12 signaling pathway. PPI analysis identified key hub genes related to sepsis and DM, and immune cell composition analysis revealed correlations between immune cells and PRDEGs. These findings indicate that the identified PRDEGs are closely associated with both sepsis and DM, suggesting their potential as molecular markers for future research into shared mechanisms underlying the two diseases.

## 1. Introduction

Sepsis and diabetes mellitus (DM) are major global health problems with high morbidity and mortality. Sepsis, characterized by a dysregulated inflammatory response to infection, is a severe condition that can rapidly progress to multiple organ dysfunction and threaten life. The economic and healthcare burden of sepsis is substantial. Millions of cases occur annually worldwide, leading to high healthcare costs and significant resource demands [1]. Similarly, DM, a chronic metabolic disorder, poses a major threat to public health. It is associated with serious complications, including cardiovascular disease, neuropathy, and kidney failure [2]. The relationship between sepsis and DM is complex. Individuals with DM are at increased risk of sepsis due to impaired immune responses and chronic inflammation [3]. Conversely, sepsis can worsen glycemic control and lead to stress hyperglycemia, which complicates the management of patients with DM [4].

Despite advances in treatment, including antibiotic therapy and supportive care, clinical outcomes remain suboptimal. This is partly due to antibiotic resistance and the complexity of patient conditions [5]. The management of DM mainly relies on glycemic control through pharmacotherapy and lifestyle modification. However, many patients still develop complications that are difficult to prevent or manage effectively [6]. Therefore, exploration of the molecular links between sepsis and DM and identification of novel therapeutic targets are warranted.

In this study, pyroptosis-related differentially expressed genes (PRDEGs) were investigated as potential targets. Pyroptosis is a form of programmed cell death that is closely associated with inflammation. Previous studies have shown that cell death pathways, including pyroptosis, may contribute to the inflammatory microenvironment in both diseases, suggesting a link with disease progression [7]. However, the specific role of pyroptosis in the pathogenesis of sepsis and DM remains unclear.

A comprehensive bioinformatics workflow was applied to identify PRDEGs in sepsis and DM. The biological pathways and signaling processes involved were analyzed. This approach aims to improve understanding of these diseases, identify candidate biomarkers, and provide a basis for future diagnostic and therapeutic studies.

## 2. Materials and methods

### 2.1. Data acquisition

**Fig 1:**
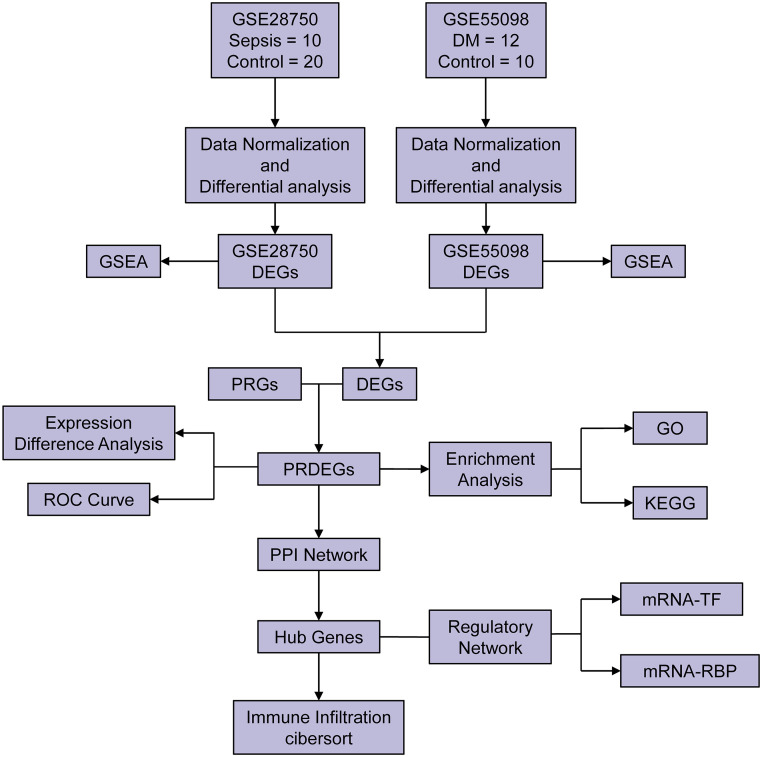
Flowchart for Comprehensive Analysis of PRDEGs. DM, diabetes mellitus; DEGs, differentially expressed genes; PRGs, pyroptosis-related genes; PRDEGs, pyroptosis-related differentially expressed genes; GSEA, gene set enrichment analysis; GO, Gene Ontology; KEGG, Kyoto Encyclopedia of Genes and Genomes; PPI, protein-protein interaction; ROC Curve, receiver operating characteristic curve; TF, transcription factor; RBP, RNA-binding protein.

The datasets analyzed in this study were obtained from the Gene Expression Omnibus (GEO) database [8] using the GEOquery package [9] (version 2.70.0) in R software. GEO is a public database from which users can freely download data for research and subsequent publication. As our study uses publicly available data, no ethical concerns or conflicts of interest exist. Specifically, two datasets were retrieved: GSE28750 (https://www.ncbi.nlm.nih.gov/geo/query/acc.cgi?acc=GSE28750) [10] related to sepsis, and GSE55098 (https://www.ncbi.nlm.nih.gov/geo/query/acc.cgi?acc=GSE55098) [11] associated with DM. The public annotation for GSE28750 indicates that samples were obtained from whole blood of sepsis patients and healthy controls, and the data were derived from a multicenter prospective critical care cohort enrolling participants at four tertiary hospitals in Australia. While GSE55098 samples were derived from peripheral blood mononuclear cells (PBMCs) of newly diagnosed type 1 diabetes patients and normal controls. Both datasets originated from Homo sapiens and were generated using the GPL570 detection platform. All samples were blood-derived specimens containing disease and control groups, fully meeting the criteria for comparative transcriptomic analysis between the two diseases. Due to consistency in species, sample type, and detection platform, these datasets were suitable for subsequent differential expression analysis and identification of common candidate genes. Comprehensive details of the datasets are provided in Table 1. All samples from the sepsis, DM, and control groups were included in subsequent analyses (Fig 1).

**Table 1:** GEO Microarray Chip Information.

	GSE28750	GSE55098
**Platform**	GPL570	GPL570
**Species**	Homo sapiens	Homo sapiens
**Tissue**	Blood	Peripheral blood
**Samples in Sepsis/DM group**	10	12
**Samples in Control group**	20	10
**Reference**	PMID: 21682927	PMID: 24796653

As a single data source may not guarantee adequate retrieval coverage and sufficient literature support simultaneously, this study constructed the pyroptosis-related gene (PRG) set by combining database retrieval from GeneCards [12] with literature screening in PubMed. In GeneCards, “pyroptosis” was used as the search keyword, retaining only protein-coding genes with relevance scores greater than 1, yielding 405 candidate PRGs. PubMed was simultaneously searched using “pyroptosis” as the primary keyword [13–23], obtaining 249 genes from published studies. During literature screening, known classical PRGs (e.g., inflammasome sensors, caspase-1/4/5/11, and gasdermin) were prioritized to ensure biological relevance to pyroptosis. The gene lists from both sources were subsequently merged and duplicates were removed, resulting in a final set of 424 PRGs for subsequent analyses. Detailed information about these genes is provided in S1 Table.

After downloading the gene expression matrices, probe IDs were mapped to corresponding gene symbols using the GPL570 platform annotation file. Datasets GSE28750 and GSE55098 were preprocessed independently. For multiple probes targeting the same gene, the arithmetic mean of expression values was calculated. Each dataset was normalized separately using the normalizeBetweenArrays function from the limma package (version 3.58.1). Data quality assessment was performed by comparing global expression distributions before and after normalization. Boxplots were used to visually inspect median expression shifts and dispersion across samples. After normalization, expression profiles in both datasets exhibited tighter clustering and more uniform dispersion compared to the pre-normalization state. No samples showed significant abnormalities; therefore, all samples were retained for subsequent analyses.

### 2.2. PRDEGs associated with sepsis and DM

Differential expression analyses were separately conducted on datasets GSE28750 and GSE55098 using the limma package (version 3.58.1). For the sepsis dataset (GSE28750), due to its large sample size and significant expression variability, |log2 fold change (logFC)| > 1 and adjusted P value < 0.05 were adopted as screening criteria. Multiple testing correction was performed using the Benjamini-Hochberg (BH) method to ensure robust biological significance. Genes with logFC > 1 were defined as upregulated, while those with logFC < −1 were considered downregulated. For the DM dataset (GSE55098), due to its smaller sample size and weaker expression differences, |logFC| > 0.5 and P value < 0.05 were applied to avoid selecting excessive genes lacking biological relevance. Differential expression results from both datasets were visualized separately using volcano plots generated by the ggplot2 package (version 3.4.4). Heatmaps depicting the expression profiles of the top 20 DEGs were created using the pheatmap package (version 1.0.12).

To identify PRDEGs common to both sepsis and DM, an intersection analysis between the DEGs from both datasets and the PRG set was conducted. A Venn diagram was constructed to illustrate overlapping relationships, enhancing the reliability and robustness of the differential expression findings.

### 2.3. GO and KEGG pathway enrichment analyses

Gene Ontology (GO) analysis is widely used for large-scale enrichment, categorizing genes into biological processes (BP), molecular functions (MF), and cellular components (CC). The Kyoto Encyclopedia of Genes and Genomes (KEGG) database [24] integrates comprehensive information on biological pathways, genomic data, diseases, and drug interactions. Functional enrichment analysis of PRDEGs via GO and KEGG databases was performed using the clusterProfiler package (version 4.10.0) [25]. To ensure rigorous enrichment criteria, adjusted P value < 0.05 and q-value < 0.25 were applied, with multiple testing correction performed again using the BH method.

### 2.4. GSEA

To evaluate overall enrichment patterns of the entire gene expression profiles within predefined pathways, gene set enrichment analysis (GSEA) [26] was separately conducted for GSE28750 and GSE55098. Genes in each dataset were sorted in descending order based on logFC values. GSEA was performed using the clusterProfiler package (version 4.10.0) [25] with a random seed set to 2022 and gene sets restricted to sizes ranging from 10 to 500 genes. The reference gene sets were obtained from the C2.cp.all.v2022.1.Hs.symbols.gmt (All Canonical Pathways) collection within the MSigDB database [27], comprising 3050 canonical pathways. Significant enrichment was defined by adjusted P value < 0.05 and FDR q-value < 0.25, with multiple testing correction using the BH method.

### 2.5. PPI network and hub gene screening

PPI networks consist of interacting proteins that play essential roles in various biological processes, including metabolism, signal transduction, gene regulation, and cell cycle progression. Investigating these interactions is crucial for understanding protein functions, elucidating biological signaling pathways, and interpreting metabolic reactions under different physiological and pathological conditions. In this study, the STRING database [28] was used to analyze protein interactions, incorporating experimentally validated and predicted associations. PRDEGs served as input to establish a PPI network, with a moderate interaction confidence threshold (interaction score ≥ 0.400). Highly interconnected clusters in this network likely represent molecular complexes performing specific biological functions. Genes exhibiting significant interactions within this network were selected as hub genes for further evaluation.

To further investigate gene functions, evaluate candidate genes, and prioritize future studies, the GeneMANIA database [29] was employed. GeneMANIA identifies genes functionally associated with query genes by integrating extensive genomic and proteomic data. Each dataset in GeneMANIA is assigned a weight reflecting its relevance to the query genes. Additionally, GeneMANIA predicts gene functions by identifying other genes likely sharing similar roles based on interaction patterns. In this study, GeneMANIA’s online tool was utilized to identify genes functionally related to the previously selected hub genes, facilitating the construction of the final PPI network.

### 2.6. Regulatory network construction

Transcription factors (TFs) regulate hub gene expression at the post-transcriptional level. To clarify these regulatory mechanisms, potential TFs interacting with the hub genes were identified using the ChIPBase database [30]. The identified mRNA-TF interactions were integrated into a regulatory network and visualized using Cytoscape software [31].

Additionally, RNA-binding proteins (RBPs) [32] significantly influence gene regulation by mediating RNA metabolism processes, such as RNA synthesis, translation control, modifications, transport, and alternative splicing. The StarBase v3.0 database [33] was used to predict RBPs interacting with hub genes. The obtained interactions were visualized as an mRNA-RBP regulatory network using Cytoscape software.

### 2.7. Differential expression and ROC curve analysis of PRDEGs

To examine differential expression patterns of PRDEGs among the sepsis, DM, and control groups in datasets GSE28750 and GSE55098, comparative expression analyses were visualized. Receiver operating characteristic (ROC) curve analyses were conducted using the pROC package (version 1.18.5) [34]. Areas under the ROC curves (AUC) were calculated to evaluate the diagnostic efficacy of PRDEGs for distinguishing sepsis and DM from controls. AUC values typically range from 0.5 to 1, with values closer to 1 indicating better diagnostic accuracy. Specifically, AUC values of 0.5–0.7 indicate poor predictive performance, 0.7–0.9 moderate accuracy, and greater than 0.9 excellent diagnostic capability.

### 2.8. Analysis of peripheral blood immune cell composition

Transcriptomic expression matrices were analyzed using the CIBERSORT algorithm, a linear support vector regression-based method, to estimate the relative composition and abundance of immune cells within complex cellular mixtures. Immune cell composition data were obtained by applying the LM22 gene signature set in CIBERSORT to datasets GSE28750 and GSE55098, retaining only cell populations with enrichment scores greater than zero. Composition ratios were presented visually using bar graphs. Differences in immune cell composition between sepsis or DM samples and their corresponding controls were visualized using the ggplot2 package (version 3.4.4). Given that samples used in this study originated from whole blood or peripheral blood mononuclear cells, the resulting data primarily reflect circulating immune cell characteristics rather than actual immune cell infiltration within tissue microenvironments. Subsequent analyses involved inter-group comparisons and correlation evaluations based on relative proportions of each immune cell subset. After identifying immune cell types with statistically significant differences, Spearman correlation analyses were performed to explore associations among immune subsets. Correlation patterns among immune cells were visualized as heatmaps using the pheatmap package (version 1.0.12). Additionally, Spearman correlation tests were conducted to examine relationships between immune cells and hub genes. Significant correlations (p < 0.05) were displayed as bubble plots generated by the ggplot2 package (version 3.4.4).

### 2.9. Statistical analysis

All data analyses and statistical processing were performed using R software (version 4.3.0). For comparisons between two independent groups, normally distributed continuous variables were analyzed using independent Student’s t-tests unless otherwise specified. When normality assumptions were violated, the Mann-Whitney U test (Wilcoxon rank-sum test) was applied. For comparisons involving three or more independent groups, the Kruskal-Wallis test was conducted. Spearman correlation analysis was used to evaluate relationships among molecular variables with different attributes. All statistical tests were two-tailed unless otherwise indicated, and results were considered statistically significant at p < 0.05.

## 3. Results

### 3.1. Technology roadmap

See Fig 1

### 3.2. Normalization of sepsis and diabetes datasets

**Fig 2:**
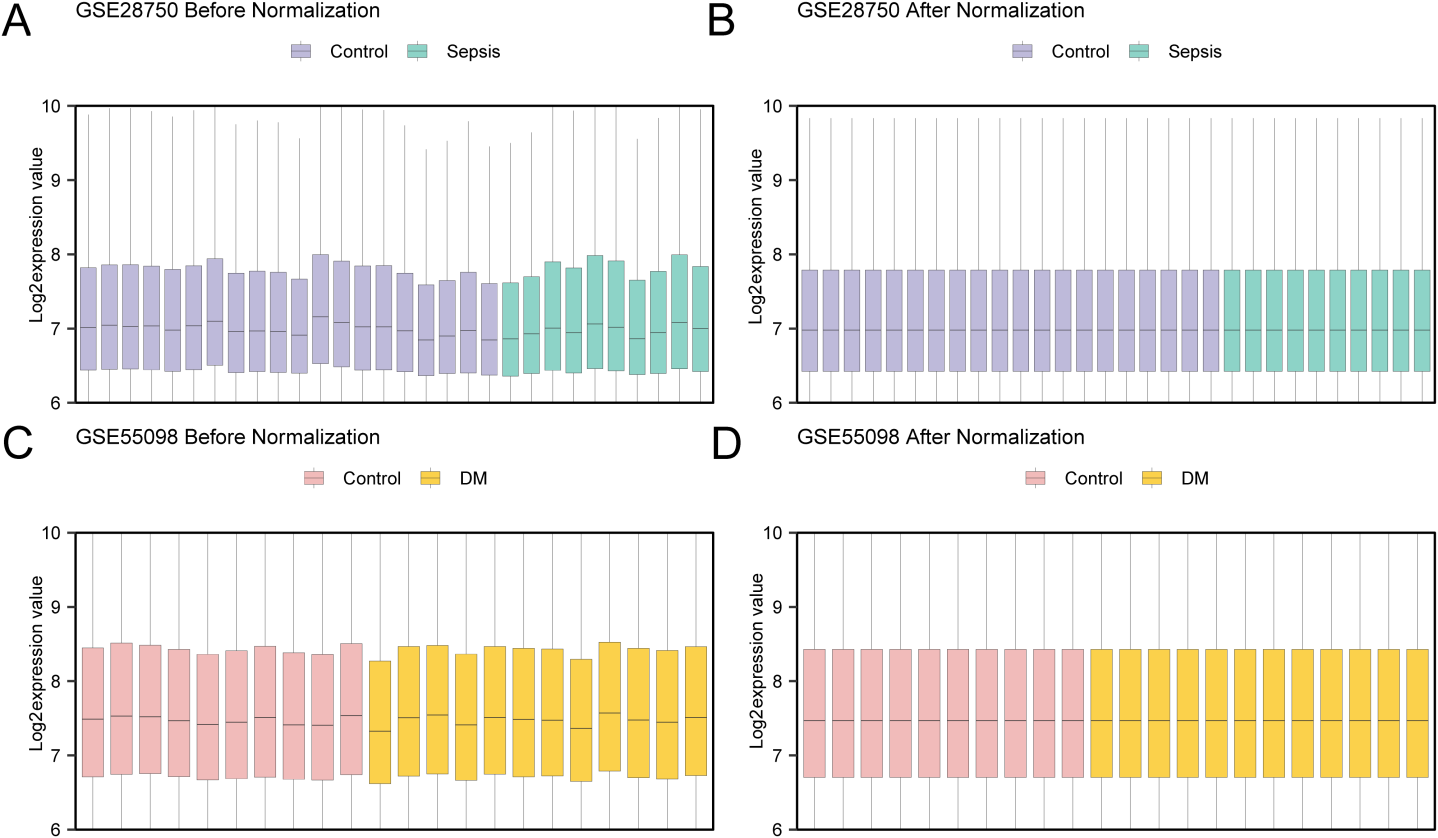
Normalization of GSE28750 and GSE55098. A-B. Boxplots of dataset GSE28750 distributions before (A) and after (B) normalization. C-D. Boxplots of dataset GSE55098 distributions before (C) and after (D) normalization.

Following annotation of probes to corresponding gene symbols based on the GPL570 platform, normalization was separately performed on datasets GSE28750 (Fig 2A-B) and GSE55098 (Fig 2C-D). The distributions of sample expression before and after normalization were compared. Boxplots demonstrated substantially more consistent median expression levels and distribution patterns among individual samples after normalization. These results confirmed satisfactory data comparability for subsequent differential expression analysis and functional enrichment analyses.

DM, diabetes mellitus. Green and purple represent the sepsis and control groups in dataset GSE28750, respectively, while yellow and pink represent the DM and control groups in dataset GSE55098, respectively.

### 3.3. PRDEGs in sepsis and DM

**Fig 3:**
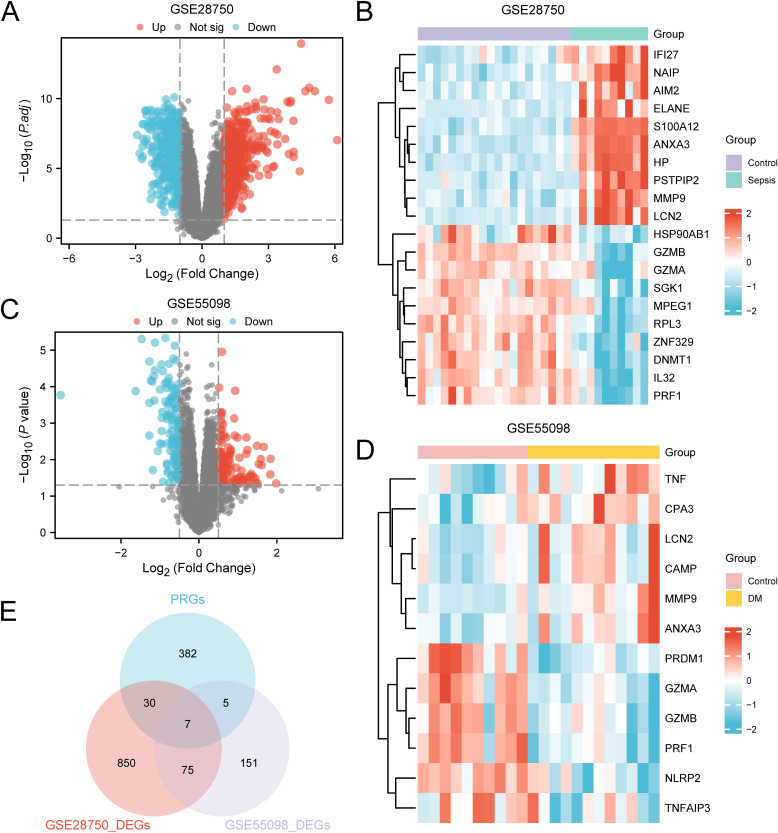
Differential Gene Expression Analysis. A-B. Volcano plot (A) and heatmap (B) of DEGs in sepsis versus control samples (dataset GSE28750). In the volcano plot, upregulated genes are marked in red, downregulated genes in blue, and genes not meeting the threshold in gray. The x-axis indicates log_2_(fold change), and the y-axis shows -log_10_(adjusted P value). The heatmap color bar indicates relative gene expression levels ranging from −2 to 2, with red indicating higher and blue indicating lower expression. C-D. Volcano plot (C) and heatmap (D) of DEGs in DM versus control samples (dataset GSE55098). In the volcano plot, upregulated genes are marked in red, downregulated genes in blue, and genes not meeting the threshold in gray. The x-axis represents log_2_(fold change), and the y-axis represents -log_10_(P value). The heatmap color bar indicates relative gene expression levels ranging from −2 to 2, with red indicating higher and blue indicating lower expression. **E.** Venn diagram illustrating the intersection of DEGs and PRGs in datasets GSE28750 and GSE55098. DM, diabetes mellitus; DEGs, differentially expressed genes; PRGs, pyroptosis-related genes.

In dataset GSE28750, after BH correction, 962 DEGs were identified using thresholds of adjusted P value < 0.05 and |logFC| > 1. Among these DEGs, 518 were upregulated and 444 were downregulated. The differential gene expression results were visualized using a volcano plot (Fig 3A), accompanied by a heatmap (Fig 3B) to illustrate expression profiles.

In dataset GSE55098, 238 DEGs were identified using thresholds of P value < 0.05 and |logFC| > 0.5. Of these, 109 genes were upregulated and 129 genes were downregulated. A volcano plot (Fig 3C) and a heatmap (Fig 3D) were generated to visually illustrate these results.

An intersection analysis was conducted between the identified DEGs from the two datasets and the PRG set, ultimately identifying seven PRDEGs: *GZMA, GZMB, MMP9, LCN2, ANXA3, PRF1,* and *CAMP*. These genes formed the basis for subsequent functional enrichment analysis, PPI network construction, and immune infiltration analysis, aiming to further evaluate shared pyroptosis-related molecular characteristics between sepsis and DM.

### 3.4. GO and KEGG pathway enrichment analysis

**Fig 4:**
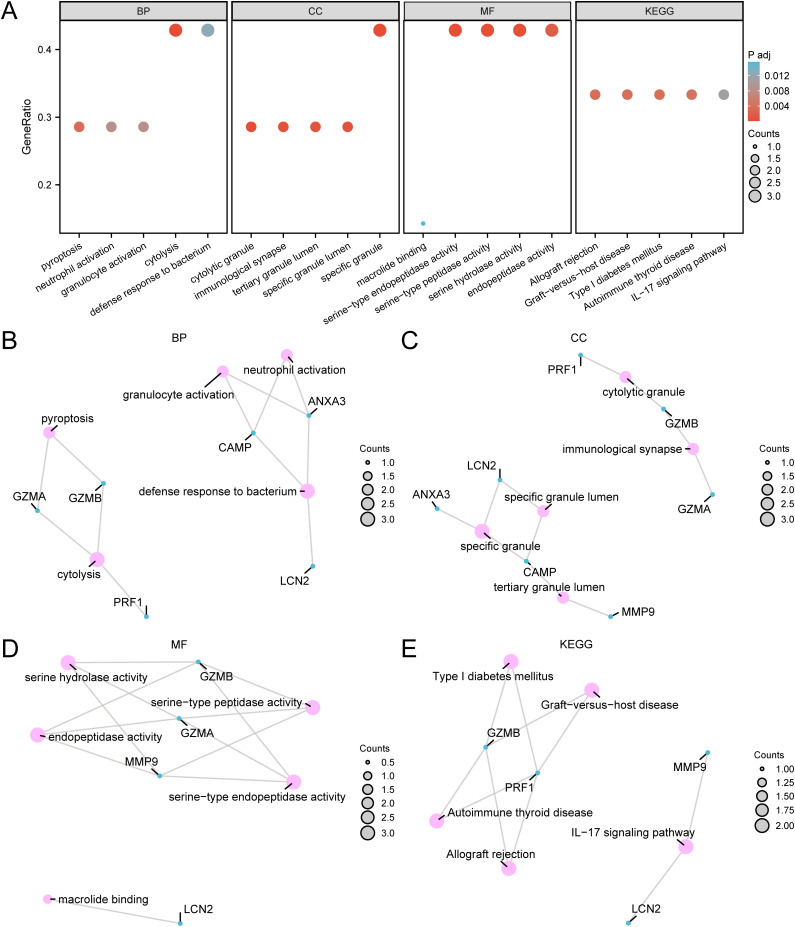
GO and KEGG Pathway Enrichment Analysis for PRDEGs. **A.** Bubble diagram summarizing GO and KEGG pathway enrichment analysis results (BP, CC, MF, and KEGG biological pathways). GO and KEGG terms are shown on the x-axis. B-E. Network diagrams showing results for BP **(B)**, CC **(C)**, MF **(D)**, and KEGG (E) enrichment analyses. Pink nodes represent enriched terms, blue nodes represent genes, and connecting lines represent relationships between terms and genes. PRDEGs, pyroptosis-related differentially expressed genes; GO, Gene Ontology; KEGG, Kyoto Encyclopedia of Genes and Genomes; BP, biological process; CC, cellular component; MF, molecular function. In the bubble plot, bubble size represents the number of genes, and bubble color indicates the adjusted P value. The color bar on the right presents the corresponding value range; colors change from red to blue, indicating increasing adjusted P value. The adjusted P value for entries shown in this figure range from approximately 1.69 × 10 ⁻ ⁵ to 0.01562.

GO and KEGG enrichment analyses were performed on the seven PRDEGs. After correction using the BH method, multiple significant terms were identified (adjusted P value < 0.05 and q-value < 0.25). Detailed results are presented in Table 2. Specifically, enriched BP terms included cytolysis, pyroptosis, granulocyte and neutrophil activation, and bacterial defense responses. Enriched CC terms consisted of specific granules, cytolytic granules, immunological synapses, tertiary granule lumen, and specific granule lumen. MF enrichment prominently included serine-type peptidase activity, serine-type endopeptidase activity, endopeptidase activity, serine hydrolase activity, and macrolide binding. KEGG enrichment revealed associations with type I DM, autoimmune thyroid disease, graft-versus-host disease, allograft rejection, and the IL-17 signaling pathway. Bubble plots were used to visually summarize the enrichment outcomes (Fig 4A).

**Table 2:** Results of GO and KEGG Enrichment Analysis for PRDEGs.

ONTOLOGY	ID	Description	GeneRatio	BgRatio	pvalue	p.adjust	qvalue
**BP**	GO:0019835	cytolysis	3/7	24/18800	6.37631E-08	1.68972E-05	7.78581E-06
**BP**	GO:0070269	pyroptosis	2/7	22/18800	2.73545E-05	0.003624465	0.001670061
**BP**	GO:0042119	neutrophil activation	2/7	41/18800	9.67756E-05	0.008443084	0.003890359
**BP**	GO:0036230	granulocyte activation	2/7	47/18800	0.000127443	0.008443084	0.003890359
**BP**	GO:0042742	defense response to bacterium	3/7	364/18800	0.0002378	0.012603401	0.005807327
**CC**	GO:0044194	cytolytic granule	2/7	13/19594	8.5174E-06	0.000153313	4.48284E-05
**CC**	GO:0042581	specific granule	3/7	160/19594	1.82588E-05	0.000164329	4.80495E-05
**CC**	GO:0001772	immunological synapse	2/7	44/19594	0.000102757	0.000616542	0.000180276
**CC**	GO:1904724	tertiary granule lumen	2/7	55/19594	0.000161003	0.000724513	0.000211846
**CC**	GO:0035580	specific granule lumen	2/7	62/19594	0.000204777	0.000737197	0.000215555
**KEGG**	hsa05330	Allograft rejection	2/6	38/8164	0.000312759	0.00374342	0.002533141
**KEGG**	hsa05332	Graft-versus- host disease	2/6	42/8164	0.000382551	0.00374342	0.002533141
**KEGG**	hsa04940	Type I diabetes mellitus	2/6	43/8164	0.000401081	0.00374342	0.002533141
**KEGG**	hsa05320	Autoimmune thyroid disease	2/6	53/8164	0.000610058	0.004270403	0.002889746
**KEGG**	hsa04657	IL-17 signaling pathway	2/6	94/8164	0.00190925	0.010691801	0.007235053
**MF**	GO:0004252	serine-type endopeptidase activity	3/7	174/18410	2.82464E-05	0.000251403	8.35687E-05
**MF**	GO:0008236	serine-type peptidase activity	3/7	191/18410	3.73145E-05	0.000251403	8.35687E-05
**MF**	GO:0017171	serine hydrolase activity	3/7	195/18410	3.96952E-05	0.000251403	8.35687E-05
**MF**	GO:0004175	endopeptidase activity	3/7	432/18410	0.000418626	0.001988473	0.000660988
**MF**	GO:0005527	macrolide binding	1/7	12/18410	0.004554566	0.015622146	0.005192957

Interactive network diagrams were constructed to illustrate relationships between significantly enriched KEGG pathways and GO terms (BP, CC, and MF) (Fig 4B-E). In these diagrams, enriched terms are depicted as nodes, with node size proportional to the number of involved genes. Edges represent molecular interactions.

### 3.5. GSEA

**Fig 5:**
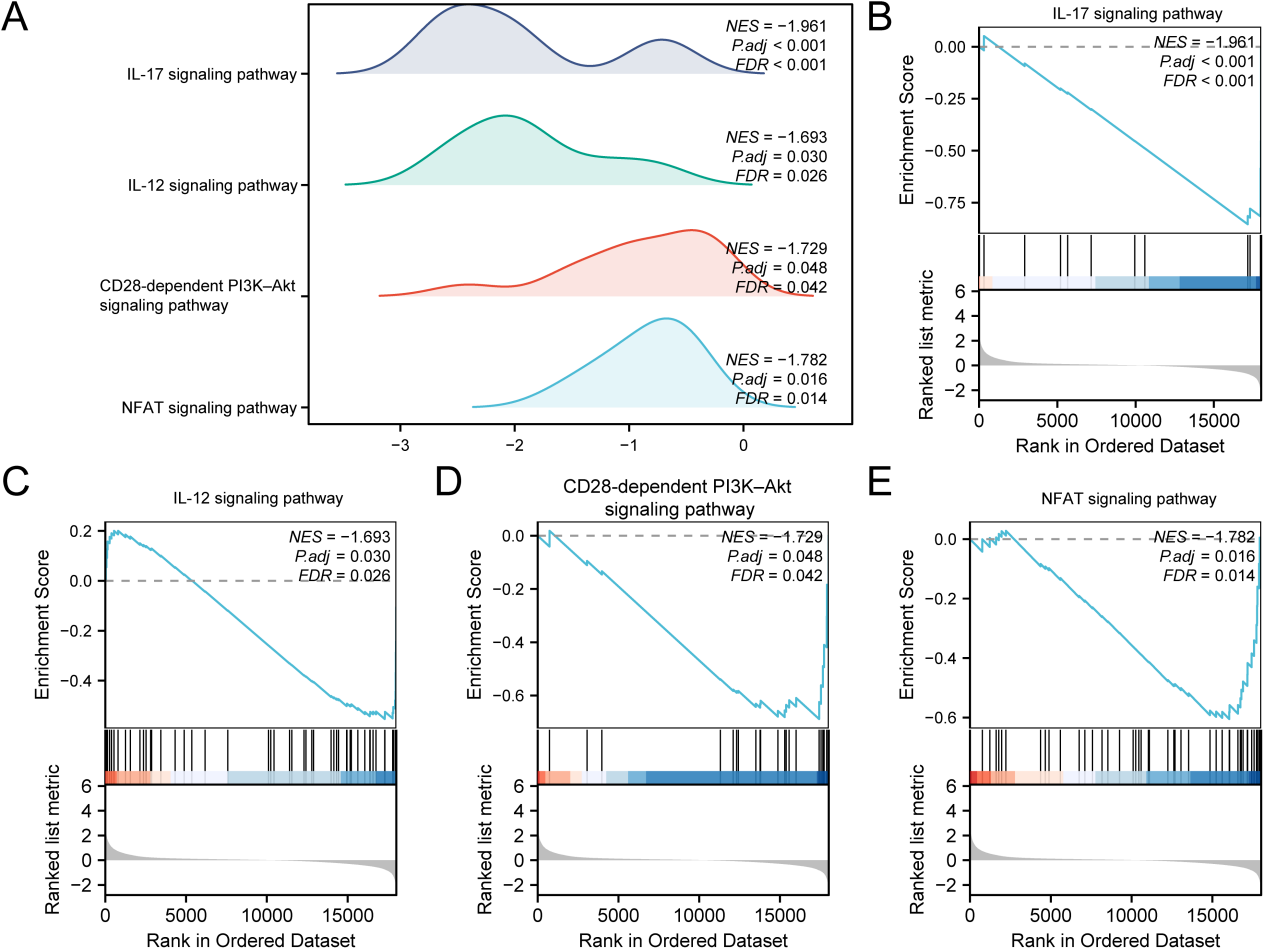
GSEA for GSE28750. **A.** Mountain plot showing four biological functions enriched in dataset GSE28750. B-E. GSEA results illustrate significant enrichment in the IL-17 signaling pathway **(B)**, IL-12-mediated signaling pathway **(C)**, CD28-dependent PI3k-Akt signaling pathway (D) and NFAT-dependent transcription pathway **(E)**. GSEA, Gene Set Enrichment Analysis.

In dataset GSE28750, GSEA was performed on the whole-genome expression profile ranked by logFC (Fig 5A). Enrichment results were corrected using the BH method, with selection criteria of adjusted P value < 0.05 and q-value < 0.25 (Table 3). Considering normalized enrichment scores (NES) and biological relevance, representative pathways were highlighted, including the IL-17 signaling pathway (Fig 5B), IL-12-mediated signaling pathway (Fig 5C), CD28-dependent PI3K-Akt signaling (Fig 5D), and NFAT-dependent transcription pathway (Fig 5E). These pathways are primarily involved in inflammatory responses, immune activation, and cellular signal transduction processes. Leading-edge genes driving these enrichments include *CXCL8, CD3D, CD8A, CD247, STAT4, TBX21, LCK, AKT1, AKT2, AKT3, PIK3R1, NFATC1, NFATC2, NFATC3,* and *FASLG*.

**Table 3:** Results of GSEA for GSE28750.

ID	Set Size	Enrichment Score	NES	pvalue	p.adjust	qvalue
**REACTOME_NEUTROPHIL_DEGRANULATION**	448	0.67919493	2.63477897	1E-10	3.9687E-08	3.4748E-08
**REACTOME_EUKARYOTIC_TRANSLATION_INITIATION**	100	−0.69685433	−2.33227258	1E-10	3.9687E-08	3.4748E-08
**REACTOME_RRNA_PROCESSING**	168	−0.646329	−2.32645809	1E-10	3.9687E-08	3.4748E-08
**REACTOME_GENERATION_OF_SECOND_MESSENGER_MOLECULES**	29	−0.86942095	−2.31469008	1E-10	3.9687E-08	3.4748E-08
**REACTOME_TRANSLATION**	248	−0.58603806	−2.1819066	1E-10	3.9687E-08	3.4748E-08
**REACTOME_EXTRACELLULAR_MATRIX_ORGANIZATION**	290	0.54050589	2.04008	1E-10	3.9687E-08	3.4748E-08
**REACTOME_PROCESSING_OF_CAPPED_INTRON_CONTAINING_PRE_MRNA**	227	−0.5512972	−2.02321786	1.1206E-10	3.9687E-08	3.4748E-08
**WP_CYTOPLASMIC_RIBOSOMAL_PROTEINS**	70	−0.71784144	−2.26589771	2.1771E-10	6.7463E-08	5.9068E-08
**REACTOME_INFLUENZA_INFECTION**	134	−0.61178875	−2.14072404	4.0422E-10	1.1134E-07	9.7485E-08
**WP_MODULATORS_OF_TCR_SIGNALING_AND_T_CELL_ACTIVATION**	60	−0.73189654	−2.23778902	3.1308E-09	7.7613E-07	6.7955E-07
**REACTOME_EUKARYOTIC_TRANSLATION_ELONGATION**	73	−0.69485029	−2.22199183	3.7746E-09	7.7976E-07	6.8273E-07
**REACTOME_NONSENSE_MEDIATED_DECAY_NMD**	94	−0.65298896	−2.15545561	3.4748E-09	7.7976E-07	6.8273E-07
**REACTOME_MRNA_SPLICING**	180	−0.55169126	−2.00144457	7.6161E-09	1.4523E-06	1.2716E-06
**BIOCARTA_CTLA4_PATHWAY**	19	−0.89725331	−2.18048563	1.1901E-08	2.1073E-06	1.845E-06
**KEGG_PRIMARY_IMMUNODEFICIENCY**	35	−0.79283957	−2.2221259	1.4815E-08	2.4484E-06	2.1437E-06
**REACTOME_DEGRADATION_OF_THE_EXTRACELLULAR_MATRIX**	138	0.60755164	2.09345144	1.7143E-08	2.6561E-06	2.3256E-06
**REACTOME_COSTIMULATION_BY_THE_CD28_FAMILY**	65	−0.69939064	−2.16126299	1.9276E-08	2.8108E-06	2.4611E-06
**WP_PATHOGENESIS_OF_SARSCOV2_MEDIATED_BY_NSP9NSP10_COMPLEX**	19	−0.89276668	−2.16958231	2.2367E-08	3.0805E-06	2.6971E-06
**REACTOME_COLLAGEN_DEGRADATION**	64	0.7261158	2.24275464	2.7934E-08	3.6446E-06	3.1911E-06
**KEGG_ANTIGEN_PROCESSING_AND_PRESENTATION**	74	−0.67140545	−2.15039085	4.0234E-08	4.9871E-06	4.3665E-06

**Fig 6:**
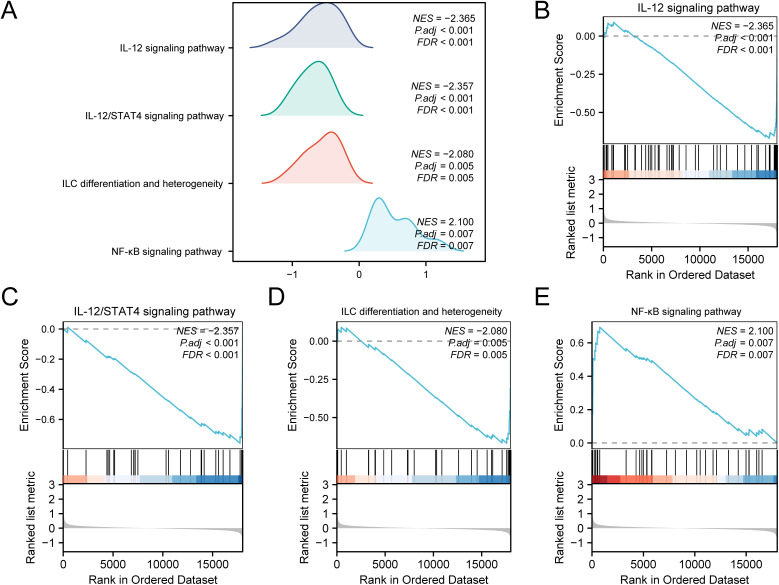
GSEA for GSE55098. **A.** Mountain plot displaying four biological functions enriched in dataset GSE55098. B-E. GSEA results demonstrate significant enrichment in the IL-12-mediated signaling pathway **(B)**, IL-12 signaling mediated by STAT4 **(C)**, Development and heterogeneity of the ILc family **(D)**, and Photodynamic therapy-induced NF-κB survival signaling **(E)**. DM: Diabetes Mellitus; GSEA, Gene Set Enrichment Analysis.

Similarly, in dataset GSE55098, GSEA was performed on the whole-genome expression profile ranked by logFC (Fig 6A). The enrichment results were corrected using the BH method (adjusted P value < 0.05 and q-value < 0.25), and detailed findings are shown in Table 4. Based on enrichment direction, NES, and relevance to the research topic, representative pathways such as IL-12-mediated signaling pathway (Fig 6B), IL-12 signaling mediated by STAT4 (Fig 6C), Development and heterogeneity of the ILC family (Fig 6D), and Photodynamic therapy-induced NF-κB survival signaling (Fig 6E) were presented. These results suggest diabetes-related expression changes are closely linked to immune regulation and abnormal inflammatory signaling. Leading-edge genes associated with these pathways mainly include *STAT4, TBX21, IL18R1, IL12RB2, LCK, IL2RG, EOMES, IFNG, GZMA, GZMB, PRF1,* as well as *MMP9, CXCL8, TNF,* and *PTGS2*.

**Table 4:** Results of GSEA for GSE55098.

ID	Set Size	Enrichment Score	NES	pvalue	p.adjust	qvalue
**REACTOME_NEUTROPHIL_DEGRANULATION**	448	0.66575831	3.00820106	1E-10	2.479E-07	2.1937E-07
**PID_IL12_2PATHWAY**	60	−0.66905995	−2.36472108	1.0327E-08	1.28E-05	1.1327E-05
**REACTOME_RESPIRATORY_ELECTRON_TRANSPORT**	93	−0.59125656	−2.25739969	4.1304E-08	3.4131E-05	3.0202E-05
**REACTOME_MITOCHONDRIAL_TRANSLATION**	91	−0.57291426	−2.18402026	1.1286E-07	6.5057E-05	5.7569E-05
**REACTOME_RESPIRATORY_ELECTRON_TRANSPORT_ATP_SYNTHESIS_BY_CHEMIOSMOTIC_COUPLING_AND_HEAT_PRODUCTION_BY_UNCOUPLING_PROTEIN S**	100	−0.56202316	−2.17824214	1.3122E-07	6.5057E-05	5.7569E-05
**WP_MODULATORS_OF_TCR_SIGNALING_AND_T_CELL_ACTIVATION**	60	−0.63370452	−2.23976109	2.222E-07	9.1806E-05	8.124E-05
**PID_IL12_STAT4_PATHWAY**	32	−0.7576291	−2.35736068	3.5025E-07	9.6475E-05	8.5372E-05
**REACTOME_ANTIMICROBIAL_PEPTIDES**	65	0.66513846	2.32558893	3.235E-07	9.6475E-05	8.5372E-05
**REACTOME_EXTRACELLULAR_MATRIX_ORGANIZATION**	290	0.43939309	1.92060565	3.3809E-07	9.6475E-05	8.5372E-05
**WP_COMPLEMENT_SYSTEM**	91	0.60477768	2.25601887	4.6621E-07	0.00011557	0.00010227
**REACTOME_TRANSLATION**	248	−0.4242726	−1.85484511	5.2068E-07	0.00011734	0.00010384
**KEGG_T_CELL_RECEPTOR_SIGNALING_PATHWAY**	107	−0.54561856	−2.14315095	6.4859E-07	0.00013399	0.00011857
**REACTOME_THE_CITRIC_ACID_TCA_CYCLE_AND_RESPIRATORY_ELECTRON_TRANSPORT**	150	−0.47638875	−1.96525577	2.068E-06	0.00039436	0.00034897
**BIOCARTA_TOB1_PATHWAY**	19	−0.79555357	−2.1758262	2.3326E-06	0.00041305	0.00036551
**PID_TCR_PATHWAY**	62	−0.60310359	−2.13784355	2.8845E-06	0.00047353	0.00041903
**KEGG_NATURAL_KILLER_CELL_MEDIATED_CYTOTOXICITY**	128	−0.49212926	−1.98422518	3.0563E-06	0.00047353	0.00041903
**WP_CANCER_IMMUNOTHERAPY_BY_PD1_BLOCKADE**	22	−0.78881981	−2.24113333	3.4579E-06	0.00047623	0.00042142
**REACTOME_IMMUNOREGULATORY_INTERACTIONS_BETWEEN_A_LYMPHOID_AND_A_NON_LYMPHOID_CELL**	124	−0.49311664	−1.98407349	3.2928E-06	0.00047623	0.00042142
**WP_TCELL_RECEPTOR_SIGNALING_PATHWAY**	88	−0.53909784	−2.03989394	3.8696E-06	0.00050489	0.00044678
**REACTOME_MRNA_SPLICING**	180	−0.44005547	−1.87022383	6.3441E-06	0.00078635	0.00069585

### 3.6 Construction of PPI network and screening of hub genes

**Fig 7:**
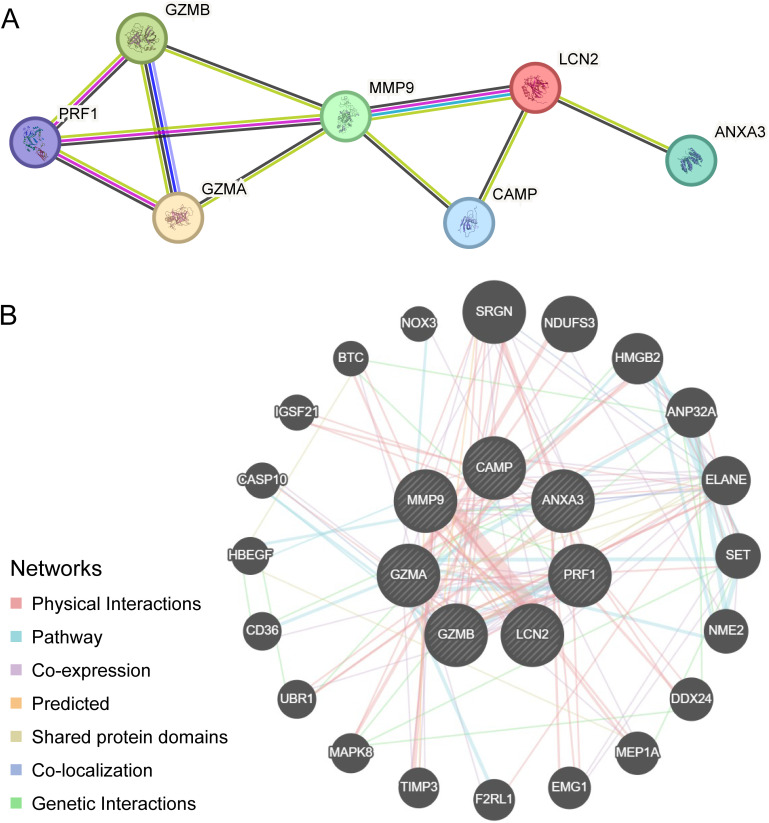
PPI Network and Hub Gene Analysis. **A.** PPI network of PRDEGs derived from the STRING database. **B.** Interaction network of hub genes and functionally related genes predicted by the GeneMANIA database. Circles represent hub genes and related genes; connecting lines indicate functional relationships. PPI, protein-protein interaction; PRDEGs, pyroptosis-related differentially expressed genes.

After constructing the PPI network of PRDEGs using the STRING database (Fig 7A), *GZMA, GZMB, MMP9, LCN2, ANXA3, PRF1,* and *CAMP* exhibited high connectivity, indicating their crucial roles in inflammatory and immune regulatory processes underlying sepsis and DM. Thus, these genes were selected as hub genes. Additionally, a functional interaction network for these seven hub genes was constructed using the GeneMANIA database (Fig 7B). The results demonstrated potential functional connections between these hub genes and various molecules involved in immune responses and cytotoxicity.

### 3.7. Construction of regulatory network

**Fig 8:**
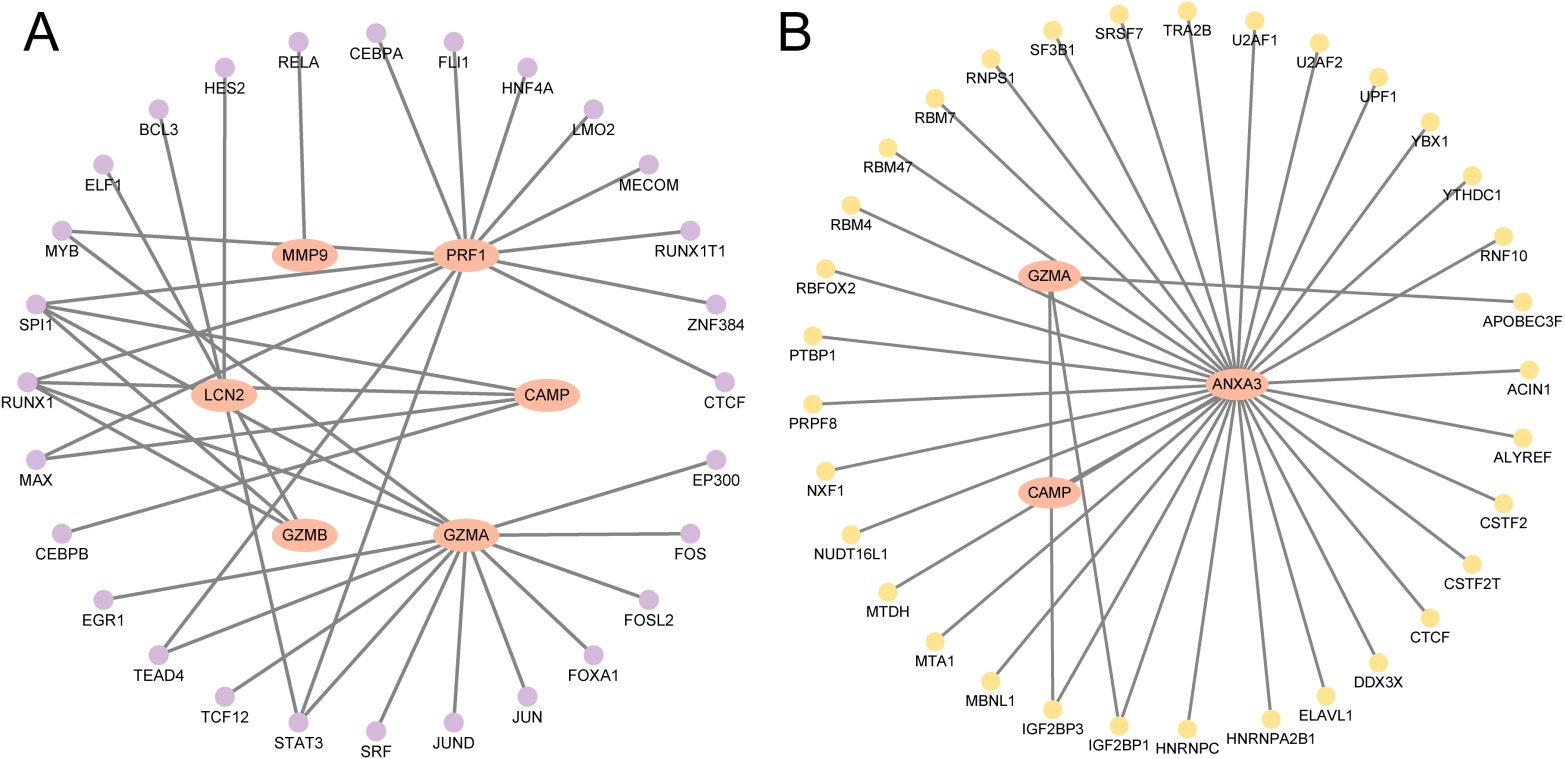
Regulatory Network of Hub Genes. A. mRNA-TF regulatory network of hub genes. B. mRNA-RBP regulatory network of hub genes. TF, transcription factor; RBP, RNA-binding protein. Orange nodes represent mRNA, purple nodes represent TFs, and yellow nodes represent RBPs.

TFs targeting hub genes were identified from the ChIPBase database. These interactions were used to construct a regulatory network comprising 28 TFs and six hub genes (*GZMA, GZMB, MMP9, LCN2, PRF1, CAMP*). This network was visualized using Cytoscape software (Fig 8A). Further details on identified TFs are summarized in S2 Table. Moreover, RBPs potentially interacting with hub genes were predicted using StarBase. A separate regulatory network consisting of three hub genes (*GZMA, ANXA3, CAMP*) and 33 RBPs was developed and visualized with Cytoscape (Fig 8B). Detailed data are provided in S3 Table. These results offer additional insights into the transcriptional and post-transcriptional regulatory mechanisms affecting these candidate genes.

### 3.8. Differential expression and ROC curve analysis of PRDEGs

**Fig 9:**
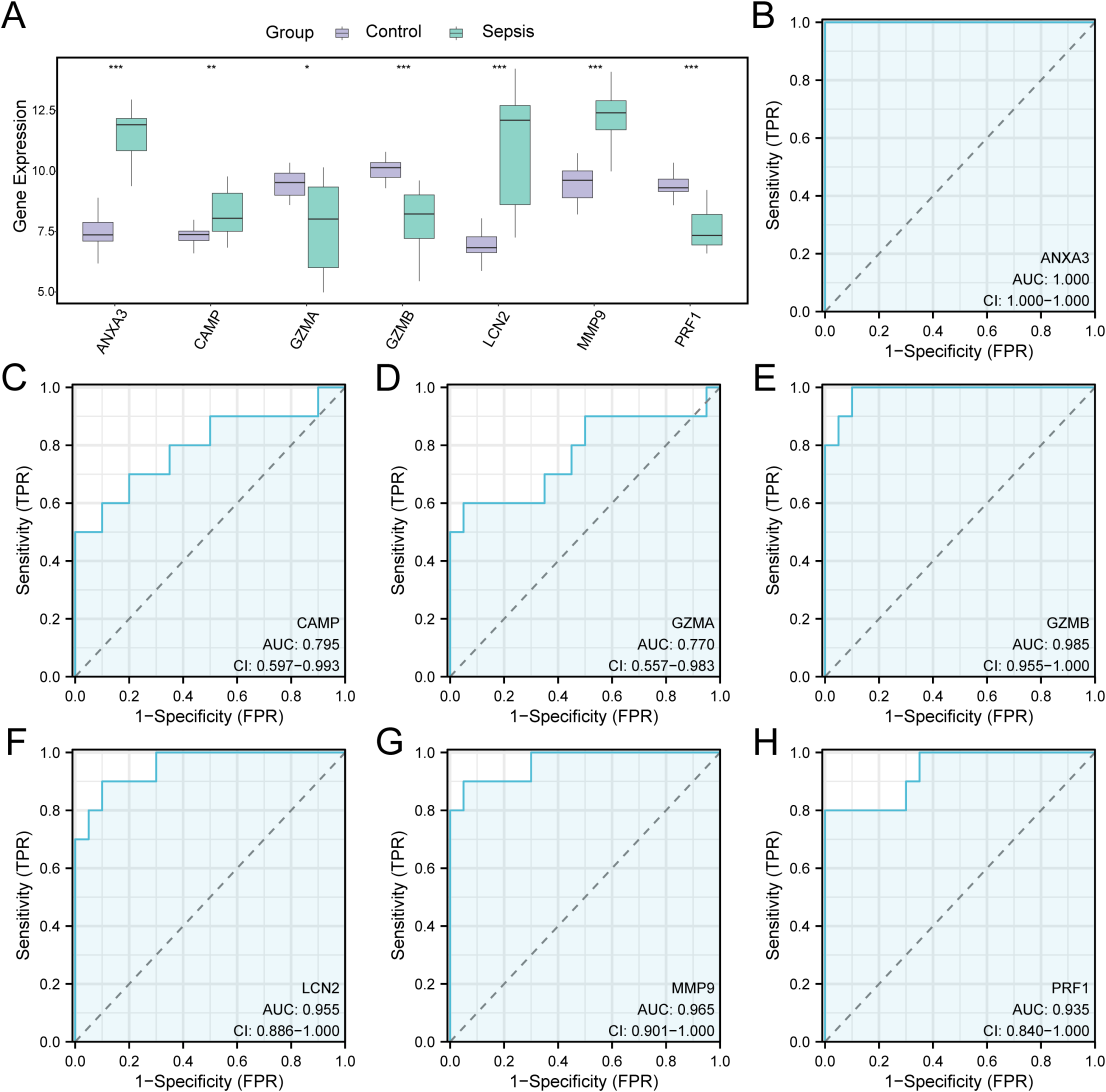
Differential Expression Validation and ROC Curve Analysis for GSE28750. **A.** Expression comparisons of PRDEGs between sepsis and control samples in dataset GSE28750. B-H. ROC curves of PRDEGs: *ANXA3* **(B)**, *CAMP* **(C)**, *GZMA* **(D)**, *GZMB* **(E)**, *LCN2* **(F)**, *MMP9* **(G)**, and *PRF1* **(H)**. *p < 0.05, significant; **p < 0.01, highly significant; ***p < 0.001, extremely significant. AUC values reflect discriminatory performance in the present datasets (0.5-0.7, low; 0.7-0.9, moderate; > 0.9, relatively high). Purple represents control samples; green represents sepsis samples. PRDEGs, pyroptosis-related differentially expressed genes; ROC, receiver operating characteristic; AUC, area under the curve; TPR, true positive rate; FPR, false positive rate.

To evaluate differential expression among the seven PRDEGs (*GZMA*, *GZMB*, *MMP9*, *LCN2*, *ANXA3*, *PRF1*, and *CAMP*) in dataset GSE28750, comparative expression plots between sepsis and control groups were generated (Fig 9A). Differential analysis revealed highly significant differences (p < 0.001) for *GZMB*, *MMP9*, *LCN2*, *ANXA3*, and *PRF1*. *CAMP* exhibited significant differences (p < 0.01), while *GZMA* showed moderate significance (p < 0.05). ROC curves assessing diagnostic performance based on these PRDEGs were generated using the pROC package (version 1.18.5) (Fig 9B-H). ROC analysis indicated moderate discriminatory performance (0.7 < AUC < 0.9) for *CAMP* and *GZMA* and relatively high discriminatory performance (AUC > 0.9) for *GZMB*, *MMP9*, *LCN2*, *ANXA3*, and *PRF1* in distinguishing sepsis from control samples.

**Fig 10:**
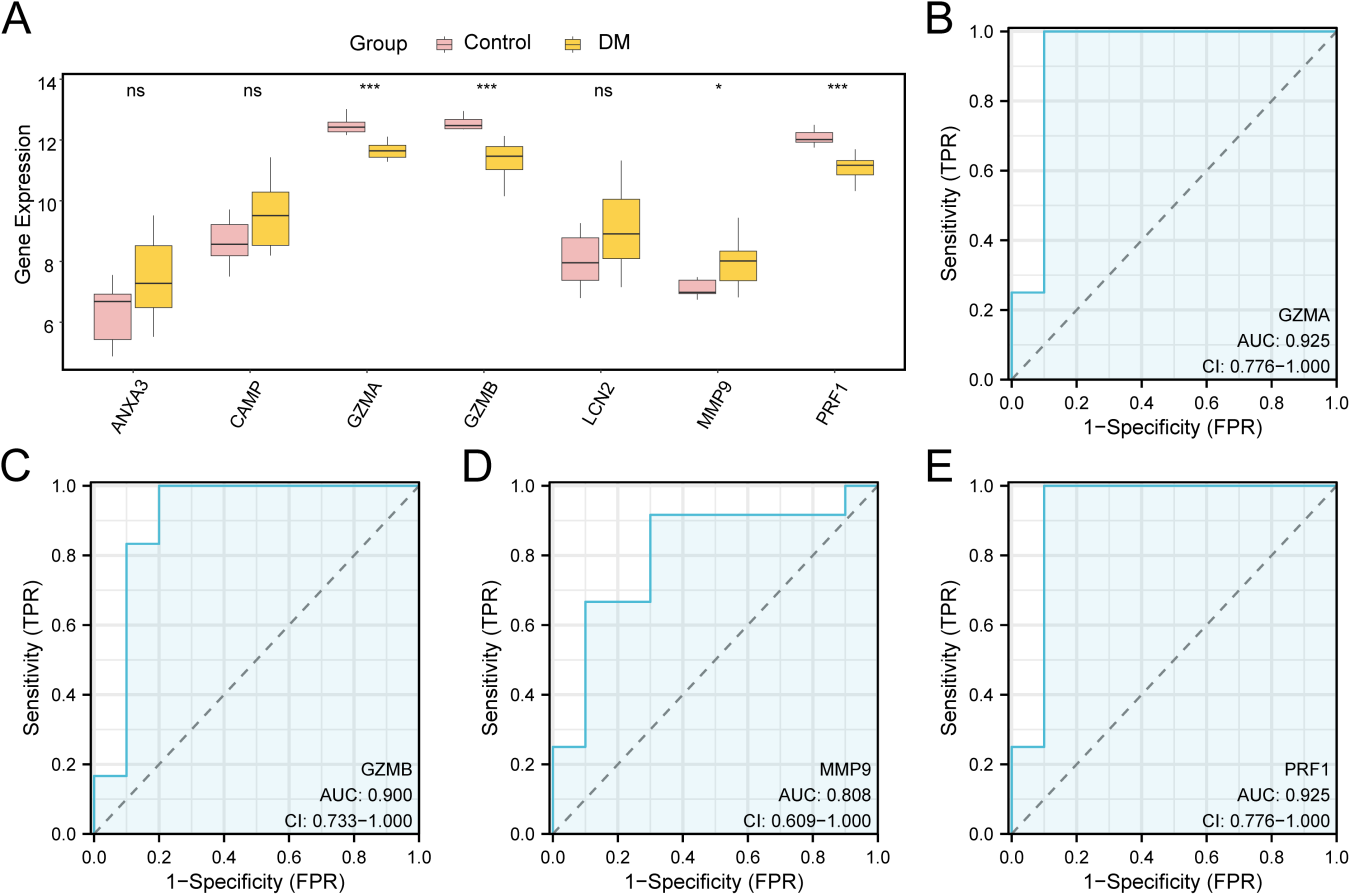
Differential Expression Validation and ROC Curve Analysis for GSE55098. **A.** Expression comparisons of PRDEGs between DM and control samples in dataset GSE55098. B-E. ROC curves of PRDEGs: *GZMA* **(B)**, *GZMB* **(C)**, *MMP9* **(D)**, and *PRF1* **(E)**. ns indicates no significance (p ≥ 0.05); *p < 0.05, significant; ***p < 0.001, extremely significant. AUC values reflect discriminatory performance in the present datasets (0.5-0.7, low; 0.7-0.9, moderate; > 0.9, relatively high). Pink represents control samples; yellow represents DM samples. DM, diabetes mellitus; PRDEGs, pyroptosis-related differentially expressed genes; ROC, receiver operating characteristic; AUC, area under the curve; TPR, true positive rate; FPR, false positive rate; ns, not statistically significant.

Similarly, to evaluate differential expression between DM and control samples in dataset GSE55098, comparative expression plots were created (Fig 10A). Differential expression analysis revealed highly significant differences (p < 0.001) for *GZMA*, *GZMB*, and *PRF1*, and moderate significance (p < 0.05) for *MMP9*. ROC curves based on PRDEG expression in GSE55098 were plotted (Fig 10B-E). ROC evaluation demonstrated moderate discriminatory performance (0.7 < AUC < 0.9) for *MMP9* and relatively high discriminatory performance (AUC > 0.9) for *GZMA*, *GZMB*, and *PRF1* in distinguishing DM from control samples.

### 3.9. Analysis of peripheral blood immune cell composition

**Fig 11:**
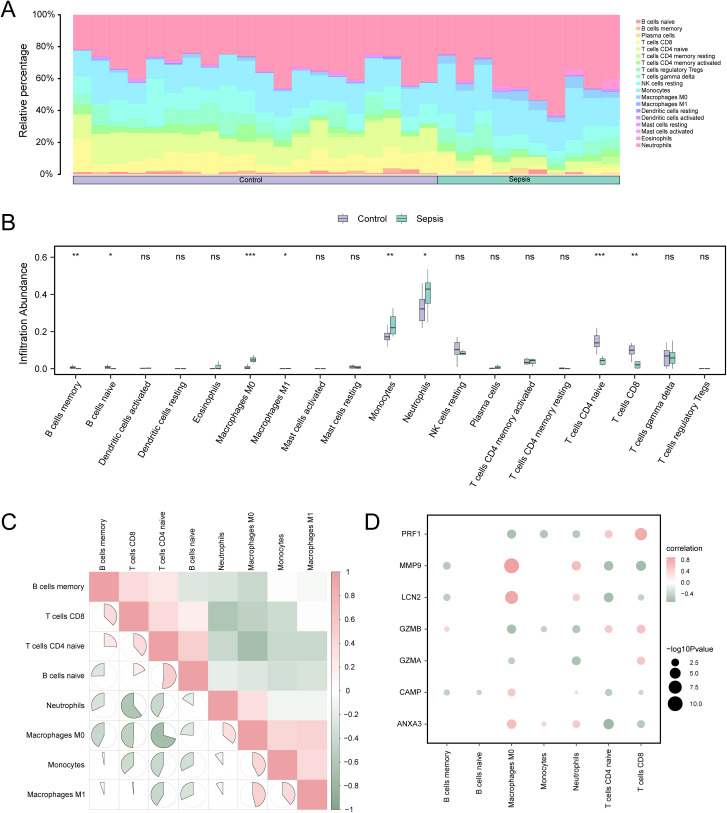
Analysis of Peripheral Blood Immune Cell Composition in GSE28750. A-B. Bar plot showing immune cell proportions (A) and group comparison plot **(B)**. **C.** Correlation heatmap of immune cell subsets. **D.** Bubble plot depicting correlations between hub genes and immune cells. The color bar on the right side of the correlation heatmap indicates the correlation coefficient *r* between immune cells, ranging from −1 to 1. Absolute correlation values *r* below 0.3 indicate weak or no correlation, values between 0.3-0.5 weak correlation, between 0.5-0.8 moderate correlation, and above 0.8 strong correlation. Green denotes negative correlation, pink positive correlation, and darker colors indicate stronger correlation intensity. Green and purple colors represent sepsis and control samples, respectively.

**Fig 12:**
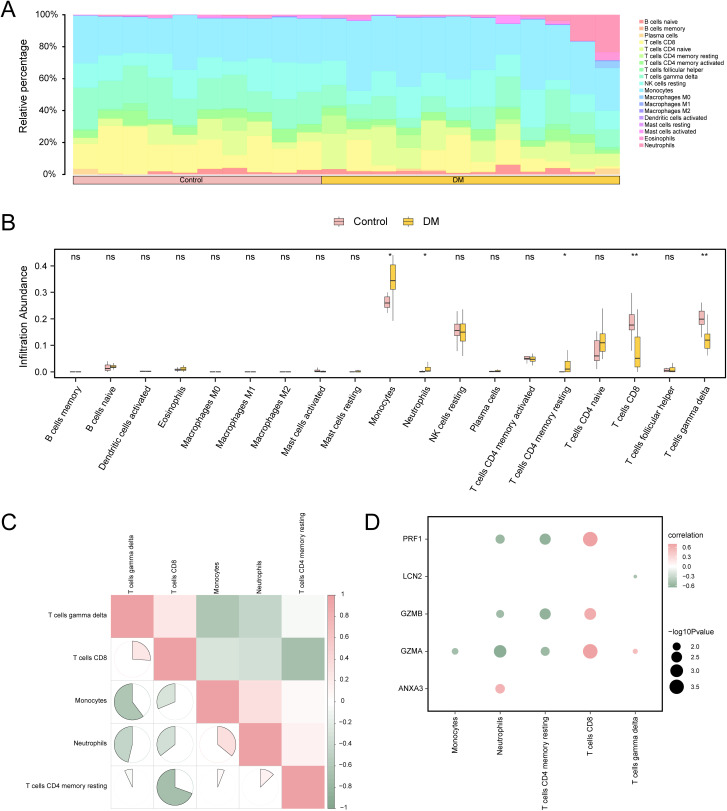
Analysis of Peripheral Blood Immune Cell Composition in GSE55098. A-B. Bar plot showing immune cell proportions (A) and group comparison plot **(B)**. **C.** Correlation heatmap of immune cell subsets. **D.** Bubble plot depicting correlations between hub genes and immune cells. DM, diabetes mellitus. The color bar on the right side of the correlation heatmap indicates the correlation coefficient *r* between immune cells, ranging from −1 to 1. Absolute correlation values *r* below 0.3 indicate weak or no correlation, values between 0.3-0.5 weak correlation, between 0.5-0.8 moderate correlation, and above 0.8 strong correlation. Green denotes negative correlation, pink positive correlation, and darker colors indicate stronger correlation intensity. DM samples are shown in yellow, control samples in pink.

The relative composition of immune cell subsets in datasets GSE28750 and GSE55098 was estimated using the CIBERSORT algorithm. Bar charts were generated to visualize the proportions of immune cells in each sample (Fig 11A, Fig 12A). Given the limited sample size and exploratory nature of this analysis, no additional sample filtering based on the deconvolution P value was performed. Instead, comparisons of immune cell proportions between different groups, and correlation analyses among immune cells and between immune cells and hub genes, were conducted using available samples. Comparative plots were then generated to highlight differences in immune cell subset composition among sepsis, DM, and control groups (Fig 11B, Fig 12B).

In dataset GSE28750, eight immune cell subsets (Monocyte, Naive B cell, Memory B cell, Naive CD4 + T cell, CD8 + T cell, Neutrophil, M0 Macrophage, and M1 Macrophage) showed statistically significant differences (p < 0.05) between sepsis and control groups. Dataset GSE55098 exhibited significant differences (p < 0.05) in four immune subsets (Neutrophil, Monocyte, Resting CD4 + memory T cell, and CD8 + T cell) between DM and control samples.

Heatmaps were created to visualize correlations among 19 immune cell subsets in each dataset (Fig 11C, Fig 12C). In dataset GSE28750, the strongest positive correlation (r = 0.524) was between Naive CD4 + T cells and Naive B cells, while the strongest negative correlation (r = −0.706) was observed between Naive CD4 + T cells and M0 Macrophages. Dataset GSE55098 showed the strongest positive correlation (r = 0.359) between Monocytes and Neutrophils, and the strongest negative correlation (r = −0.695) between Resting CD4 + memory T cells and CD8 + T cells.

Bubble plots were generated to illustrate significant correlations between immune cell populations and selected hub genes (Fig 11D, Fig 12D). In dataset GSE28750, hub gene *MMP9* displayed a notable positive correlation with M0 Macrophages (r = 0.886, p < 0.05), whereas *ANXA3* exhibited a strong negative correlation with Naive CD4 + T cells (r = −0.686, p < 0.05). In dataset GSE55098, *GZMA* showed a marked positive correlation with CD8 + T cells (r = 0.702, p < 0.05) and a significant negative correlation with Neutrophils (r = −0.646, p < 0.05).

## 4. Discussion

Sepsis and DM are significant global health challenges, placing considerable burdens on patients and healthcare systems. Sepsis, characterized by a systemic inflammatory response to infection, exhibits high morbidity and mortality rates, significantly impacting patient health and healthcare resources [35]. Similarly, DM, a chronic metabolic disorder affecting glucose homeostasis, is associated with various complications that reduce quality of life and increase healthcare costs [36]. The interaction between these two conditions complicates clinical management, as patients with DM have an increased risk of infections, including those potentially leading to sepsis [37]. Understanding the molecular mechanisms underlying these diseases is essential for developing more effective therapeutic strategies and improving patient outcomes.

In this study, we focused on PRDEGs in sepsis and DM. Previous research established that pyroptosis, a form of programmed cell death associated with inflammation, plays a critical role in various inflammatory diseases [38]. However, the relationships between PRDEGs and these diseases, and their specific association patterns, remain unclear. Through comprehensive bioinformatics analysis, this study aimed to identify PRDEGs shared by sepsis and DM, explore their biological functions and associations with the diseases, and provide clues for potential biomarkers.

By separately identifying DEGs in sepsis and DM and intersecting them with the predefined PRG set, this study identified shared PRDEGs. *GZMA* and *GZMB*, serine proteases in cytotoxic lymphocytes that induce apoptosis, showed elevated expression in sepsis and DM, indicating roles in inflammation and cell death [39]. The significant upregulation of *MMP9* observed here suggests its involvement in tissue damage and inflammation, aligning with previous findings linking *MMP9* to adverse outcomes in these diseases [40]. *LCN2*, an acute-phase protein linked to inflammation and immune response [41], showed differential expression, highlighting its potential as a biomarker for disease severity, particularly in metabolic disorders. Altered expression of *ANXA3* suggests dysregulation of inflammation and apoptosis, consistent with prior studies [42]. *PRF1*, crucial for NK and CTL cytotoxicity, shows therapeutic potential. Upregulated *CAMP* expression indicates a compensatory response to infection, emphasizing its host-defense role in sepsis and DM. This association was reported for the first time in our study. Notably, not all candidate genes analyzed are core effectors of the classical inflammasome-dependent pyroptosis pathway. *GZMA, GZMB*, and *PRF1* are more typically associated with cytotoxic lymphocyte-mediated killing [43,44], whereas *MMP9, LCN2, ANXA3*, and *CAMP* primarily mediate neutrophil activation, granule release, inflammation amplification, and innate immune responses [45–47]. Pyroptosis itself is not an isolated signaling event but interconnected with cytotoxic responses, inflammatory mediator release, and immune amplification processes [48,49]. Therefore, these genes should be interpreted as components of a pyroptosis-related inflammatory network rather than core inflammasome pathway elements. Such results suggest pyroptosis-related abnormalities in sepsis and DM involve interactions beyond the classical inflammasome axis, extending to cytotoxic immune responses and broader inflammatory effector programs.

Enrichment analyses revealed that shared PRDEGs primarily participate in inflammatory responses, cytotoxicity, and immune regulation. KEGG analysis showed significant enrichment in the IL-17 signaling pathway, while GSEA demonstrated prominent transcriptional alterations in the IL-12-related pathways. These results may indicate pyroptosis’s role in sepsis and DM extends beyond cell death alone, involving inflammatory signal amplification and persistent immune imbalance, consistent with previous findings [50,51]. Pyroptosis involves the release of proinflammatory mediators, promoting downstream inflammation pathways. The IL-17 signaling pathway closely relates to neutrophil recruitment, inflammatory cytokine release, and tissue inflammation propagation [52]. Pyroptosis and IL-17 signaling may therefore reinforce each other during sustained inflammation. Conversely, IL-12-related signaling primarily regulates T cell and natural killer cell-mediated immune responses. Alterations in these pathways may disrupt cytotoxic molecule expression and immune function balance [53]. Combined with the key PRDEGs identified in this study (*GZMA, GZMB, PRF1*), the dysregulation of IL-12 pathways may provide exploratory clues for altered cytotoxic immune status linked to inflammatory changes in both sepsis and DM [54]. Collectively, these enrichment results suggest that PRDEGs may be associated with inflammatory and immune-related pathways in sepsis and DM, although the underlying mechanisms require further validation. This study provides valuable insights from the perspective of inflammatory and immune imbalance, improving our understanding of the potential shared molecular mechanisms underlying these two complex diseases. Furthermore, it establishes a foundation for future in-depth investigations targeting these pathways and candidate genes.

To further investigate potential interactions and regulatory relationships among candidate genes at a systems level, a PPI network and TF/RBP regulatory networks were constructed using publicly available databases. Within the PPI network, *GZMA, GZMB, MMP9, LCN2, ANXA3, PRF1,* and *CAMP* were identified as hub genes, highlighting their potential roles in the molecular crosstalk between sepsis and DM. These findings align with the studies conducted by Chen et al [55–59]. These genes do not exist independently; rather, they form a tightly interconnected functional module. STRING and GeneMANIA analyses indicated their associations with an additional 20 proteins involved in common biological processes. Network enrichment results suggest that these hub genes participate in several potentially shared pathways, including abnormal immune activation, extracellular matrix remodeling, and antimicrobial defense, all of which characterize both diseases.

The construction of transcriptional and post-transcriptional regulatory networks provides crucial insights into the upstream regulatory mechanisms of these hub genes. This information is essential for understanding their abnormal expression and evaluating their specificity as biomarkers. In this study, 28 TFs targeting six hub genes (*GZMA, GZMB, MMP9, LCN2, PRF1,* and *CAMP*) were identified, clarifying regulatory pathways potentially disrupted in disease states. For instance, experiments by Kumagai et al. demonstrated that ubiquitin-specific protease 7 (USP7) regulates IL-4-induced phosphorylation of STAT3, thereby influencing granzyme production during Th2 differentiation [60]. Similarly, an independent network of 33 RBPs interacting with *GZMA*, *ANXA3*, and *CAMP* was established, suggesting additional post-transcriptional regulatory mechanisms affecting mRNA stability, localization, and translation [61]. Specific abnormalities in these RBPs during disease may regulate hub gene expression independently of transcriptional changes, creating dynamic biomarkers possibly specific to disease stages [62]. This dual regulatory context enhances the diagnostic specificity of these markers, as their expression patterns reflect disease-related regulatory programs rather than general stress responses. Therefore, the highly connected genes identified in the PPI and TF/RBP networks serve as initial clues for functional associations and mechanistic hypotheses in sepsis and DM, guiding future research. However, experimental studies and rigorous statistical evaluations are still required to confirm these genes as core regulatory elements and clarify their biological significance.

ROC analyses in this study utilized the same public datasets employed for candidate gene screening. Several PRDEGs demonstrated favorable discriminatory ability between case and control groups in the datasets analyzed, indicating preliminary discriminatory potential. Notably, *GZMB* and *PRF1* consistently exhibited high discriminatory performance in both sepsis and DM analyses. Granzymes (*GZMA*, *GZMB*) and perforin *(PRF1*) are established mediators of cytotoxic lymphocyte activity [43,44] and are associated with various inflammatory diseases [63,64]. Their upregulation in both sepsis and DM may reflect shared immune dysregulation and tissue injury pathways. Nevertheless, their preliminary discriminatory potential requires further confirmation via validation in independent cohorts and rigorous evaluations. Without such external verification, these genes cannot be regarded as clinically validated diagnostic biomarkers.

Peripheral blood immune cell composition analysis revealed significant differences in immune profiles between the two datasets and highlighted interactions among immune cells in sepsis and DM, providing a reliable approach to exploring immune-related associations in these diseases. For instance, the strong positive correlation between naive B cells and naive CD4 + T cells in the GSE28750 dataset suggests a synergistic effect that may enhance adaptive immune responses [65]. Conversely, the negative correlation between naive CD4 + T cells and M0 macrophages implies potential interactions affecting inflammatory responses and tissue repair processes [66]. In the GSE55098 dataset, the negative correlation between CD8 + T cells and neutrophils indicates that increased CD8 + T cell levels may correspond to reduced neutrophil activity, influencing inflammation resolution and overall immune status [67]. Additionally, this study identified associations between hub genes (e.g., *GZMA, MMP9, ANXA3*) and specific immune cells. For example, *GZMA* showed a significant positive correlation with CD8 + T cells, consistent with the findings of Nguyen et al. [68]. These results suggest that these genes might serve as potential biomarkers for sepsis and DM. Regarding the results related to Macrophages M0 and Macrophages M1, we interpret their biological meaning as transcriptional programs similar to the signature profiles of these macrophage subsets, which likely reflect state changes in monocytes/myeloid cells rather than directly equating to tissue-resident macrophage infiltration or polarization status. However, given the limited sample size, FDR correction was not performed, which may lead to a potential risk of false positives. Peripheral blood transcriptome analysis, the LM22 gene set, and deconvolution algorithms may be influenced by transcriptional reprogramming in immune cells. Therefore, using tissue-specific samples, single-cell RNA sequencing, and more precise immune cell subset markers may further clarify the dynamic changes of immune cells and the roles of key genes in disease progression.

This study presents an exploratory bioinformatics analysis based on public transcriptome data. Its primary focus is not to propose novel inflammation-related genes, but to identify commonly differentially expressed candidate genes in the contexts of both sepsis and diabetes under the analytical framework of pyroptosis. Furthermore, we performed an integrated analysis of the potentially shared inflammatory and immune regulatory characteristics between the two diseases by combining functional enrichment, interaction networks, and immune cell composition analyses. Several genes, including *GZMB*, *MMP9*, *PRF1*, and *LCN2*, have been previously reported to be associated with inflammatory activation or immune responses. However, systematically characterizing them as shared pyroptosis-related molecular signatures of sepsis and diabetes helps elucidate their potential common biological basis from a cross-disease perspective, and provides candidate clues for future validation studies targeting shared mechanisms. Notably, as this study was based on transcriptomic and computational analyses, the inferred pathway relationships should be interpreted as exploratory associations rather than direct evidence of causal molecular mechanisms. Nevertheless, this study still has several limitations: First, all findings were obtained via bioinformatics approaches, lacking validation in independent external cohorts and adequate experimental evidence from cellular and animal models. In addition, despite using the same platform for human samples, the two GEO datasets differed in experimental workflows, data production and cohort features. We conducted separate normalization and differential analyses to obtain overlapping PRDEGs, with no cross-dataset merging or batch effect correction performed. Hence, the identified genes may be influenced by technical variations and cohort heterogeneity. Further studies with rigorous control assays, large-scale independent cohorts with full clinical data and comprehensive experimental validation are needed to verify the stability and biological specificity of our results and clarify the associated molecular mechanisms.

## 5. Conclusion

In conclusion, this study identified seven PRDEGs (*GZMA*, *GZMB*, *MMP9*, *LCN2*, *ANXA3*, *PRF1*, and *CAMP*) through bioinformatics analyses. These PRDEGs are closely associated with inflammatory responses, immune regulation, and pyroptosis-related pathways, providing insights into shared molecular features of sepsis and DM. However, as this analysis is based on public databases, the findings represent associations that require additional experimental validation and confirmation using independent cohorts to establish their biological relevance.

## Supporting information

S1 Table Pyroptosis-related genes. (CSV)

S2 Table Details of the TF regulatory network. (CSV)

S3 Table Details of the RBP regulatory network. (CSV)
